# Brachial radiculopathy with intact central nervous system imaging following carbon monoxide poisoning: A case report

**DOI:** 10.1016/j.cnp.2025.07.004

**Published:** 2025-07-26

**Authors:** Zhiyong Lin, Jierong Mo, Peiyi Liu, Zhiquan Li, Ran Zhan, Jun Jiang, Tianen Zhou

**Affiliations:** a Guangdong Medical University, Zhanjiang, Guangdong 524000, China; b Department of Emergency, The First People’s Hospital of Foshan, Foshan, Guangdong 528000, China

**Keywords:** Brachial Radiculopathy, Carbon Monoxide Poisoning, Peripheral Nerve Injury, Electrophysiological Monitoring, Hyperbaric Oxygen Therapy

## Abstract

**Objective:**

To report and analyze a unique case of selective brachial plexopathy following carbon monoxide poisoning without central nervous system involvement.

**Methods:**

Clinical examination, laboratory tests, neuroimaging, serial electrophysiological studies, and functional assessments were performed on a 25-year-old female presenting with severe left upper extremity weakness after CO exposure. Treatment included hyperbaric oxygen therapy, steroid therapy, and rehabilitation.

**Results:**

Following carbon monoxide poisoning, the patient developed complete left upper extremity paralysis with selective C5-C7 motor deficits and preserved sensory function. Brain MRI showed no abnormalities. Electrophysiological studies confirmed preganglionic radiculopathy with reduced motor nerve amplitudes and normal sensory conduction. Ultrasonography demonstrated C5-C7 nerve root swelling. Following hyperbaric oxygen therapy, corticosteroids, and rehabilitation, motor function gradually recovered with distal grip strength reaching 86% of the unaffected side at 120-day follow-up.

**Conclusions:**

This case demonstrates an uncommon manifestation of carbon monoxide poisoning characterized by isolated brachial radiculopathy with preserved central nervous system function on neuroimaging. These findings expand the recognized spectrum of carbon monoxide neurotoxicity.

**Significance:**

This case expands our understanding of CO neurotoxicity beyond the traditional basal ganglia paradigm, emphasizing the importance of peripheral nervous system assessment even when central neuroimaging is normal.

## Highlights

• CO poisoning caused isolated C5-C7 radiculopathy with normal brain MRI, challenging CNS-focused paradigms.
• Motor axons more vulnerable than sensory due to metabolic demands; positioning during unconsciousness amplified CO toxicity.
• Peripheral nerve assessment crucial in CO poisoning even with normal neuroimaging to detect nerve root injury.

## 1 Introduction

Carbon monoxide (CO) poisoning is traditionally associated with central nervous system damage, particularly affecting the basal ganglia and cerebral white matter. While central manifestations are well-documented, peripheral nervous system involvement remains underreported. Most literature describes peripheral neuropathies occurring alongside evident central damage, with isolated peripheral nerve injury being exceptionally rare. We present an unusual case of severe brachial radiculopathy following CO exposure in which brain imaging remained completely normal. This case challenges the conventional neuroimaging-focused approach to CO poisoning and highlights the importance of comprehensive peripheral neurological assessment even when central structures appear unaffected.

## 2 Case report

A 25-year-old female was found unconscious in a left lateral decubitus position on the bathroom floor after carbon monoxide exposure from a gas leak in a closed bathroom. The patient was in a deep coma (Glasgow Coma Scale 3) for approximately 30 min following rescue, with gradual improvement in consciousness over the subsequent 2 h. After 3.5 h of exposure, the patient presented with flaccid paralysis of the left upper limb (Medical Research Council [MRC] grade 0 for deltoid/biceps muscles, grade 1 for wrist extensors) and oxygen saturation of 82 % without supplemental oxygen.Clinical examination revealed selective motor deficits with preserved sensation to light touch, vibration, and proprioception in the C5-C7 dermatomes. Deep tendon reflexes were absent (grade 0) in the left biceps and brachioradialis, while triceps reflex was diminished (grade 1 + ) on the standard 0–4 + reflex grading scale.The right upper extremity showed normal strength, sensation, and reflexes throughout all tested distributions.

**Fig. 1:**
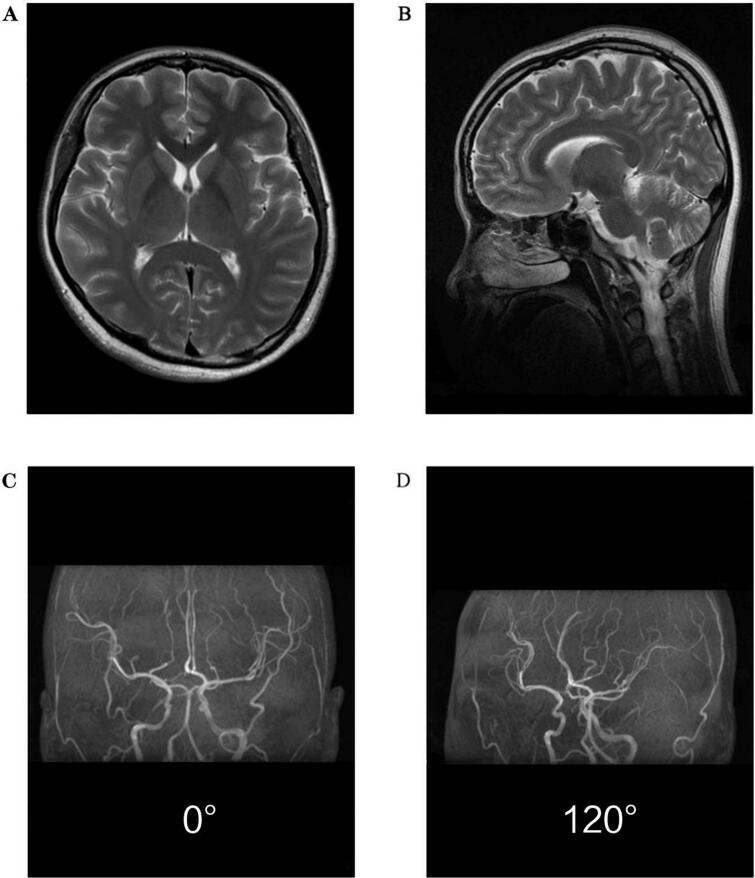
Neuroimaging findings in acute carbon monoxide poisoning. (A) Axial T2-weighted image (basal ganglia level): The head of the caudate nucleus, putamen, and posterior limb of the internal capsule demonstrate normal signal intensity without characteristic hyperintense lesions.(B) Sagittal T2-weighted image (central sulcus level): Uniform signal intensity is observed in the primary motor cortex and subcortical white matter, with no evidence of edema or necrosis.(C) Magnetic resonance angiography (MRA), anteroposterior projection (0°): The major arterial branches of the Circle of Willis (anterior cerebral artery [ACA], middle cerebral artery [MCA], and posterior communicating artery [PComA]) demonstrate normal course and patency.(D) MRA, oblique projection (120°): Focal luminal narrowing is noted in the A1 segment of the right ACA (arrow), consistent with congenital vascular developmental variation unrelated to acute toxic injury. Note: Despite severe clinical manifestations of carbon monoxide poisoning, no characteristic neuroimaging alterations were detected in susceptible gray matter predilection sites or cerebrovascular structures, contrasting distinctly with the observed brachial radiculopathy. This imaging-pathological dissociation suggests potential limitations of conventional MRI in detecting early neurotoxic damage.

Delayed carboxyhemoglobin measurement revealed a level of 2.3 % after high-flow oxygen therapy. Laboratory investigations on admission showed mild elevation of inflammatory markers: white blood cell count 10.43 × 10^9^/L (reference range: 4.0–10.0 × 10^9^/L), C-reactive protein 4.12 mg/L (reference range: <3.0 mg/L), and procalcitonin 0.95 ng/ml (reference range: <0.25 ng/ml). Brain MRI (T2-FLAIR sequence) showed no abnormalities in the basal ganglia or cortical structures (Fig. 1). Magnetic resonance angiography (MRA) was performed to exclude vascular causes of selective neural dysfunction and to assess potential CO-induced cerebrovascular changes, which showed normal vascular anatomy except for an incidental congenital A1 segment narrowing unrelated to the acute presentation.

Electromyography at 72 h post-exposure demonstrated selective motor axonal damage: left radial nerve compound muscle action potential (CMAP) amplitude of 3.0 mV (normal ≥ 4.0 mV), musculocutaneous nerve CMAP of 4.4 mV, F-wave persistence of 54.5 %, with all sensory nerve action potentials (SNAPs) showing normal amplitude and conduction velocity, presenting a typical motor-sensory dissociation characteristic of preganglionic radiculopathy(Table 1). Needle electromyography performed on day 11 revealed acute denervation changes with fibrillation potentials and positive sharp waves in the left deltoid, biceps, and extensor carpi radialis muscles, while muscles innervated primarily by sensory distributions (brachioradialis and first dorsal interosseous) showed normal spontaneous activity. F-wave studies demonstrated prolonged latencies and reduced persistence, further supporting proximal nerve root involvement.

**Table 1:** Dynamic Evolution of Electrophysiological Findings in Carbon Monoxide-Induced Brachial Radiculopathy.

**Time Point**	**Motor Nerve Conduction**	**Sensory Nerve Conduction**	**F-wave Studies**	**Needle EMG**	**Imaging Findings**
**Day 3**	Left radial nerve CMAP: 3.0 mV (Normal ≥ 4.0 mV) Left musculocutaneous CMAP: 4.4 mV Conduction velocity: Normal	All SNAPs normal: • Median nerve • Ulnar nerve • Superficial radial nerve • Lateral antebrachial cutaneous nerves	Left median nerve: • Persistence: 54.5 % • Latency: Normal	Selective motor axonal damage demonstrated Sensory potentials preserved Motor-sensory dissociation pattern	Brain MRI: Normal No basal ganglia lesions
**Day 11**	Left radial CMAP: 3.0 mV Left musculocutaneous: Mildly reduced Left median/ulnar: Mildly reduced	All sensory studies: Persistently normal Amplitude and velocity within normal limits	Left median: 54.5 % Left ulnar: 50.0 % Prolonged latencies noted	Acute denervation changes: • Deltoid: Fibs(+), PSWs(+) • Biceps: Fibs(+), PSWs(+) • Ext. carpi radialis: Fibs(+), PSWs(+) Normal activity: Brachioradialis, 1st DI	*Not performed*
**Day 19**	*Not examined in detail*	*Not examined in detail*	*Not examined*	*Not examined*	**Ultrasonography:** C5-C7 nerve root swelling Fusiform enlargement (67 mm^2^) Loss of fascicular structure
**Day 28**	Left radial CMAP: 3.7 mV ↑ Left musculocutaneous: 4.0 mV Left axillary CMAP: 4.4 mV **Wrist extensors: MRC Grade 3**	Left median/ulnar: Normal conduction Maintained throughout recovery	Left median nerve: 63.6 % ↑ Progressive improvement noted	Reduced fibrillation activity **Early reinnervation potentials** in distal muscle groups Deltoid: Still no contraction	*Not performed*
**Day 56**	Left radial CMAP: 4.3 mV ↑ Left musculocutaneous: 4.8 mV Left axillary CMAP: 5.0 mV **Near-normal amplitude recovery**	All sensory nerves: Normal • Median, ulnar • Superficial radial • Lateral antebrachial cutaneous	Left median: 68.8 % ↑ Left ulnar: 80.0 % ↑ Substantial recovery	*Not examined in detail*	**Ultrasonography:** Resolution of nerve root swelling Cross-sectional area: 42 mm^2^ Improved fascicular definition
**Day 120**	Near-complete radial nerve recovery **Distal grip strength: 86 % of unaffected side** Proximal functions: MRC Grade 5 with mild residual weakness	Complete sensory recovery All modalities normal	F-wave persistence normalized	Good reinnervation in distal muscle groups **Distal-to-proximal recovery pattern confirmed**	*Not performed*

Abbreviations and Notes: CMAP: Compound Muscle Action Potential; SNAP: Sensory Nerve Action Potential; EMG: Electromyography; MRC: Medical Research Council muscle strength grading; Fibs: Fibrillation potentials; PSWs: Positive sharp waves; DI: First dorsal interosseous; ATA: Atmospheres absolute.

Technical specifications: Nerve conduction studies performed with standard surface electrodes, skin temperature 32–34 °C. Normal radial nerve CMAP ≥ 4.0 mV. F-wave persistence > 70 % considered normal. Ultrasonography performed with 12–15 MHz transducer.

Color coding: Red indicates abnormal values (e.g., reduced CMAP amplitude); green indicates normal values; orange indicates improvement trends from previous measurements.

Treatment protocol: Hyperbaric oxygen therapy (2.4 ATA × 17 sessions), methylcobalamin (1500 μg/day), methylprednisolone (80 mg/day × 5 days).

Key finding: Motor-sensory dissociation pattern consistent with preganglionic radiculopathy affecting C5-C7 nerve roots. ↑ indicates improvement from previous measurement.

**Fig. 2:**
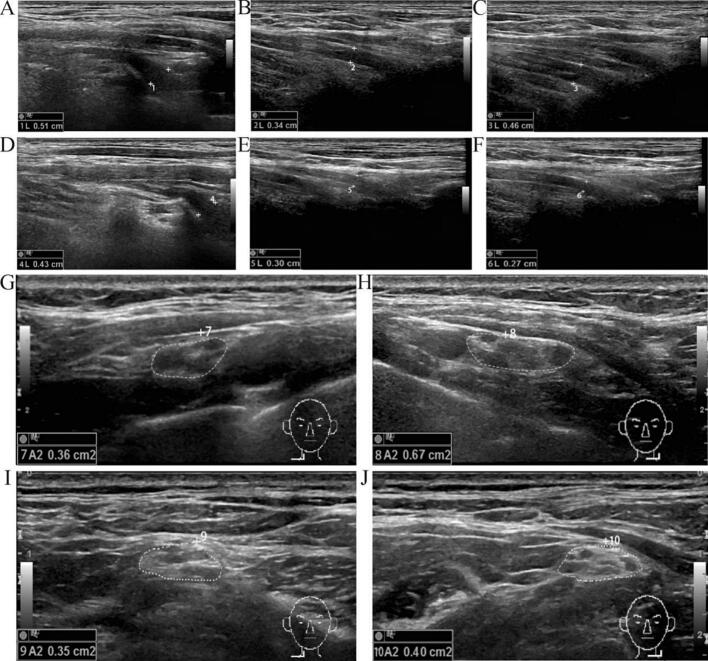
Dynamic ultrasonographic evolution of brachial plexus nerve roots following carbon monoxide poisoning. (A-C) Acute phase (Day 19) longitudinal ultrasonographic images of left C5, C6, and C7 nerve roots: Demonstrate marked nerve root swelling and thickening with reduced echogenicity and loss of normal fascicular architecture. Measured nerve root thicknesses are 5.1 mm (C5), 3.4 mm (C6), and 4.6 mm (C7), respectively. (D-F) Acute phase (Day 19) longitudinal ultrasonographic images of right C5, C6, and C7 nerve roots: Serving as the unaffected contralateral control, the right-sided nerve roots display normal echogenicity and preserved fascicular structure, with measured thicknesses of 4.3 mm (C5), 3.0 mm (C6), and 2.7 mm (C7), respectively. Bilateral comparison reveals obvious swelling and thickening of the left-sided nerve roots. (G-H) Acute phase (Day 19) axial ultrasonographic comparison: The left brachial plexus nerve roots (G) show significant fusiform swelling with blurred fascicular architecture and heterogeneous echogenicity. The C5-C7 nerve root complex demonstrates a cross-sectional area of approximately 67 mm^2^. The right brachial plexus nerve roots (H) maintain normal morphology with a cross-sectional area of approximately 36 mm^2^. The left-to-right cross-sectional area ratio of 1.86 indicates marked asymmetric swelling. (I-J) Recovery phase (Day 56) axial ultrasonographic comparison: The left brachial plexus nerve roots (I) show partial structural recovery with gradually improving fascicular definition and enhanced echo texture, though residual swelling persists with a cross-sectional area of approximately 42 mm^2^. The right brachial plexus nerve roots (J) maintain normal morphology with a cross-sectional area of approximately 35 mm^2^. The left-to-right cross-sectional area ratio decreased to 1.20, demonstrating significant recovery trends. Technical specifications: Ultrasonographic examinations were performed using a 12–15 MHz high-frequency linear array transducer with the patient positioned supine and the neck in mild extension. Measurements were obtained under standardized conditions with appropriate probe pressure and gain settings.

Ultrasound examination on day 19 revealed fusiform swelling of the left C5-C7 nerve roots (67 mm^2^ with blurred fascicular architecture and heterogeneous echogenicity)(Fig. 2A-H). The swelling pattern was uniformly distributed throughout the nerve root segments rather than focal in nature. After intervention with hyperbaric oxygen therapy (17 sessions at 2.4ATA), mecobalamin (1500 μg/d), and methylprednisolone (80 mg/d for 5 days), follow-up electromyography on day 28 showed improvement in radial nerve CMAP to 3.7 mV and wrist extensor strength to MRC grade 3, though the deltoid muscle still showed no active contraction (axillary nerve CMAP 4.4 mV). Repeat needle EMG demonstrated reduced fibrillation activity and emergence of early reinnervation potentials in distal muscle groups. Dynamic ultrasound monitoring showed resolution of C5-C7 nerve root swelling by day 56 (cross-sectional area 42 mm^2^) with F-wave persistence recovering to 80.0 %(Fig. 2I–J). At final follow-up (120 days post-exposure), a paradoxical functional recovery was observed: distal grip strength reached 86 % of the unaffected side with nearly complete radial nerve function recovery, while proximal shoulder joint strength recovered to MRC grade 5 but with residual mild weakness during complex activities.The complete temporal evolution of electrophysiological findings from acute injury through recovery is summarized in Table 1, demonstrating the characteristic progression from initial motor axonal damage to gradual functional recovery.

## 3 Interpretation

This case presents a rare manifestation of carbon monoxide (CO) poisoning characterized by severe brachial nerve root pathology with complete preservation of central nervous system function. Unlike previous reports focusing on basal ganglia lesions (Rose et al., 2017, Liu et al., 2024), this patient demonstrated complete dissociation between peripheral nerve injury and central nervous system protection.

The exposure conditions described in this case provide crucial clues for understanding the injury mechanism. The patient remained unconscious in a left lateral decubitus position within an enclosed bathroom for approximately 30 min, with deep coma (Glasgow Coma Scale score of 3) indicating severe CO exposure, while oxygen saturation dropping to 82 % reflected severe tissue hypoxia. These specific exposure conditions created a unique pathophysiological environment: the systemic metabolic toxicity of CO constituted the fundamental injury mechanism, while prolonged left lateral positioning during unconsciousness amplified this toxic effect through hemodynamic alterations. Specifically, sustained positioning reduced arterial perfusion to the left brachial plexus(Sawyer et al., 2000), making this region more vulnerable to CO-induced cellular respiratory dysfunction. Autonomic nervous system suppression during deep coma further compromised vascular autoregulation (ter Laan et al., 2013), superimposing local ischemic factors onto the foundation of systemic metabolic stress.Critically, this positioning effect functioned as a hemodynamic modifier rather than direct mechanical compression. Multiple lines of evidence support this metabolic rather than mechanical injury mechanism.

The most remarkable feature supporting a metabolic mechanism is the selective motor dysfunction with complete sensory preservation observed in this case. Electrophysiological examination showed sensory nerve action potentials (SNAPs) maintained normal amplitude and conduction velocity, while motor fibers demonstrated significant injury, with left radial nerve compound muscle action potential (CMAP) amplitude reduced to 3.0 mV, suggesting substantial motor axonal loss.The pathophysiological basis for this selective vulnerability lies in the differential metabolic demands of motor and sensory fiber types. Motor axons (Aα fibers) have larger diameters (8–12 μm) with sodium–potassium pump density at nodes of Ranvier significantly higher than sensory fibers (Grider et al., 2023; Rasband & Peles, 2015), and their ATP demands per unit length markedly exceed those of sensory axons (Aδ fibers, 3–5 μm) (Zang & Marder, 2021). When CO inhibits cytochrome *c* oxidase, high-energy-demand motor axons are the first to suffer from ATP depletion (Alonso et al., 2003). Motor axons in the C5-C7 nerve root region contain the highest density of large-diameter fibers, with mitochondrial density approximately 1.7 times that of distal nerves (Perkins & Ellisman, 2011), showing extreme susceptibility to CO-induced cytochrome oxidase inhibition. In contrast, sensory fibers demonstrate greater resistance to metabolic stress (Hofmeijer et al., 2013).

Additional evidence further establishes this metabolic injury mechanism while excluding mechanical compression. First, the motor-sensory dissociation pattern described above contradicts the typical presentation of mechanical injuries such as compartment syndrome, which usually affects both motor and sensory functions simultaneously. Second, comprehensive MRI evaluation revealed normal cervical spine anatomy, excluding structural compression from hematoma, disc herniation, and foraminal stenosis. Third, ultrasound examination on day 19 demonstrated uniform fusiform swelling of C5-C7 nerve roots, rather than the focal, asymmetric changes characteristic of mechanical compression (Mackinnon, 2002).

The temporal evolution pattern provides crucial chronological evidence supporting this metabolic mechanism. Electrophysiological dysfunction within 72 h reflected acute-phase energy depletion, when cytochrome oxidase inhibition blocked ATP generation. Needle electromyography on day 5 showing acute denervation changes (fibrillation potentials and positive sharp waves) marked the collapse of axoplasmic transport systems. Ultrasound examination on day 19 revealing fusiform swelling of C5-C7 nerve roots (67 mm^2^) confirmed morphological changes due to abnormal neurofilament protein accumulation. This uniformly distributed swelling pattern, combined with the systematic temporal progression, established the “energy depletion-axonal destruction” cascade injury mechanism: CO exposure led to ATP depletion and electrophysiological dysfunction within 72 h, followed by axoplasmic transport system collapse and abnormal neurofilament protein accumulation, with day 19 ultrasound confirming morphological changes during this progressive process.

Having established the metabolic nature of the injury mechanism, a critical question emerges: why was the damage strictly limited to C5-C7 nerve roots while sparing other cervical levels? This anatomical selectivity requires multi-level explanation that combines structural vulnerability with vascular supply patterns. Anatomically, C5-C7 nerve roots demonstrate unique anatomical vulnerability, lacking adequate ligamentous protective structures in the extraforaminal segment, making them more susceptible to metabolic injury (Zhao et al., 2020). In this case, the preserved sensory function further suggests that this anatomical vulnerability primarily affects motor fibers. From a vascular perspective, the blood supply to C5-C7 nerve roots originates from a watershed zone between the vertebral artery system and ascending cervical artery branches (Arslan et al., 2018, Gofur and Singh, 2025), creating inherently unstable perfusion that faces the dual challenge of impaired oxygen delivery and utilization during CO poisoning. The patient's specific left lateral positioning further exacerbated left-sided perfusion deficiency, explaining why only the left side was affected despite systemic CO exposure.

Having explained the local anatomical and vascular factors that made C5-C7 nerve roots vulnerable, we must address a broader paradox: why did the central nervous system remain completely unaffected while peripheral nerves suffered severe damage? This central-peripheral dissociation reveals fundamental differences in how these neural compartments respond to CO toxicity.The complete preservation of the central nervous system contrasts sharply with severe peripheral nerve injury, reflecting fundamental differences between these two neural compartments when facing CO toxicity. Brain MRI (T2-FLAIR sequences) was completely normal in this case, including the basal ganglia region traditionally vulnerable to CO injury, suggesting that the central nervous system possesses stronger protective mechanisms. Central nervous system resistance stems from multiple protective mechanisms: basal ganglia neurons can obtain alternative energy substrates through lactate metabolic coupling networks constructed by astrocytes (Zheng and Wang, 2018), while this region establishes complex redundant vascular networks through lenticulostriate arteries, enhancing hypoxic tolerance (Wei et al., 2021). In contrast, the affected brachial nerve roots in this case relied solely on low-density endoneurial microvascular blood supply, with these endothelial cells being particularly sensitive to CO-induced oxidative stress, and the lack of epineurial protection directly exposing axons to the toxic environment (Usami et al., 2022, Chiu, 2011).

Beyond primary metabolic injury, secondary inflammatory responses play important roles in disease evolution. Laboratory examinations showed mild elevation of inflammatory markers, suggesting controlled secondary inflammatory responses rather than primary inflammatory disease, consistent with the pattern of local tissue injury. The fusiform nerve root swelling and heterogeneous echogenicity observed on day 19 ultrasound likely reflect sterile inflammatory responses triggered by damage-associated molecular patterns (DAMPs) released from metabolically injured axons (Corrigan et al., 2016).

The recovery pattern demonstrated in this case shows significant spatiotemporal heterogeneity, further validating the metabolic injury mechanism. Distal radial nerve axons have relatively small diameters (approximately 3.44 μm) (Chentanez et al., 2010) and can rapidly rebuild neuromuscular connections through collateral sprouting (An et al., 2015), explaining why wrist extensor strength recovered to MRC grade 3 by day 28. In contrast, large-diameter axons of the proximal axillary nerve require longer regeneration distances and delayed synaptic reconstruction, resulting in significantly delayed deltoid muscle recovery. This imbalanced recovery pattern closely correlates with different metabolic demands determined by axonal diameter, with the low metabolic requirements of small-diameter axons making them more likely to rebuild function after injury. Notably, despite muscle strength recovery to grade 5 at final follow-up (120 days post-poisoning), the patient still experienced mild fatigue during complex proximal shoulder joint activities, suggesting possible subclinical changes in corticospinal tract microstructure.

The success of therapeutic interventions in this case provides additional validation for the injury mechanism. Hyperbaric oxygen therapy (2.4 ATA × 17 sessions) promoted neural repair through dual mechanisms: inhibiting mitochondrial lipid peroxidation in the acute phase (Palzur et al., 2008) and upregulating PGC-1α-mediated axonal mitochondrial biogenesis during recovery (Hsu et al., 2022). Electrophysiological improvement on day 28 (radial nerve CMAP increased from 3.0 mV to 3.7 mV) coincided with the treatment timeline. Short-term methylprednisolone therapy (80 mg/day × 5 days) reduced nerve root edema by decreasing TNF-α expression (Xiang et al., 2022), with dynamic ultrasound monitoring confirming nerve root cross-sectional area reduction from significant swelling on day 19 to 13.2 mm^2^ on day 56. These specific treatment responses provided indirect but strong support for the metabolic injury mechanism.

This case reveals a previously unrecognized dimension of CO poisoning neurotoxicity, demonstrating that severe peripheral nerve injury can occur with completely normal central nervous system function. The occurrence of this phenomenon required precise convergence of anatomical susceptibility (unique vulnerability of C5-C7 nerve roots), metabolic vulnerability (high energy demands of motor axons), and hemodynamic impairment (local ischemia due to left lateral positioning), forming a multifactorial interactive injury pattern.

## 4 Conclusion

This case reports a rare manifestation of carbon monoxide poisoning: severe brachial nerve root pathology with completely normal central nervous system imaging. The patient presented with a motor-sensory dissociation pattern of selective C5-C7 nerve root injury, with gradual recovery of neurological function following aggressive treatment. This case challenges the traditional understanding of carbon monoxide poisoning as predominantly involving central nervous system injury, demonstrating that severe peripheral nerve damage can occur with normal brain imaging. Clinicians managing carbon monoxide poisoning patients should conduct comprehensive peripheral nervous system assessments even when routine neuroimaging is negative, to avoid missing potential nerve root or peripheral nerve injuries.

## 5 Limitations

While this case report provides valuable insights into CO-induced Brachial Radiculopathy, several limitations should be acknowledged. The single-case design limits generalizability, though it serves as an important clinical alert.

## Disclosure Statement

This paper is new. Neither the entire paper nor any part of its content has been published or has been accepted elsewhere. The authors declare no competing interests.

## Author Contributions

TE Z and JR M prepared and wrote the article. ZQ L, LC M and WG X was directly involved in the management of the patients. LY L, PY L, ZH H and LY J were responsible for the collection and organization of the literature. J J revised the manuscript and acted as corresponding authors. All authors contributed to the article and approved the submitted version.

## Ethical approval

The study was reviewed and approved by the Ethics Committee of The First People's Hospital of Foshan. Written informed consent was obtained from the patient for the publication of this case report and any accompanying images, with the understanding that efforts would be made to conceal her identity while maintaining scientific integrity. All authors have given their consent for the manuscript to be published.

## Consent for publication

All authors have given their consent for the manuscript to be published.

## Data availability

The data that support the findings of this study are available.

## Funding

This work was supported by The Basic and Applied Basic Research Fund of Guangdong (Regional Cooperation Fund Project, 2022) [grant number No. 2022A1515140033], The Medical Research Fund of Guangdong [grant number No. A2023178] and The 14th Five-Year HighLevel Medical Key Specialty Project of Foshan. The funder had no role in study design, data collection and analysis, decision to publish, or preparation of the manuscript.

## Declaration of Competing Interest

The authors declare that they have no known competing financial interests or personal relationships that could have appeared to influence the work reported in this paper.
